# Primary care practitioners' priorities for improving the timeliness of cancer diagnosis in primary care: a European cluster-based analysis

**DOI:** 10.1186/s12913-023-09891-w

**Published:** 2023-09-16

**Authors:** Ana Luisa Neves, Magdalena Esteva, Robert Hoffman, Michael Harris

**Affiliations:** 1 Global Digital Health Unit, Department of Primary Care and Public Health, Imperial College London, London, UK; 2 Centre for Health Technology and Services Research, Department of Community Medicine, Information and Health Decision Sciences, University of Porto, Porto, Portugal; 3 Unit of Research, Majorca Department of Primary Care, Balearic Islands Health Research Institute (IdISBa), Palma, Illes Balears Spain; 4 Department of Family Medicine, Faculty of Medicine, Tel Aviv University, Tel Aviv, Israel; 5 College of Medicine and Health, University of Exeter, Exeter, UK; 6 Institute of Primary Health Care (BIHAM), University of Bern, Bern, Switzerland

**Keywords:** Primary health care, Cancer, Early diagnosis, Consultation and referral, Delivery of health care

## Abstract

**Background:**

Diagnosing cancer at an early stage increases the likelihood of survival, and more advanced cancers are more difficult to treat successfully.

Primary care practitioners (PCPs) play a key role in timely diagnosis of cancer. PCPs’ knowledge of their own patient populations and health systems could help improve the planning of more effective approaches to earlier cancer recognition and referral.

How PCPs act when faced with patients who may have cancer is likely to depend on how their health systems are organised, and this may be one explanation for the wide variation on cancer survival rates across Europe.

**Objectives:**

To identify and characterise clusters of countries whose PCPs perceive the same factors as being important in improving the timeliness of cancer diagnosis.

**Methods:**

A cluster analysis of qualitative data from an online survey was carried out. PCPs answered an open-ended survey question on how the speed of diagnosis of cancer in primary care could be improved. Following coding and thematic analysis, we identified the number of times per country that an item in a theme was mentioned. *k*-means clustering identified clusters of countries whose PCPs perceived the same themes as most important. Post-hoc testing explored differences between these clusters.

**Setting:**

Twenty-five primary care centres in 20 European countries. Each centre was asked to recruit at least 50 participants.

**Participants:**

Primary care practitioners of each country.

**Results:**

In all, 1,351 PCPs gave free-text answers. We identified eighteen themes organising the content of the responses. Based on the frequency of the themes, *k*-means clustering identified three groups of countries. There were significant differences between clusters regarding the importance of: access to tests (*p* = 0.010); access to specialists (*p* = 0.014), screening (*p* < 0.001); and finances, quotas & limits (*p* < 0.001).

**Conclusions:**

Our study identified three distinct clusters of European countries within which PCPs had similar views on the factors that would improve the timeliness of cancer diagnosis. Further work is needed to understand what it is about the clusters that have produced these patterns, allowing healthcare systems to share best practice and to reduce disparities.

## Background

Improving cancer diagnosis at an early stage, by diagnosing it more quickly and earlier, is a common aim of European healthcare providers [1–5]. This is because more advanced cancers are more difficult to treat successfully [1, 6] and, for many cancers, the likelihood of survival is related to the stage of disease at diagnosis [7, 8]. There is evidence that longer times before diagnosis and treatment increase cancer mortality [9–15]. However, cancer survival rates vary widely across Europe [16]. Data from the fifth cycle of the European Cancer Registry-based Study on Survival and Care of Cancer Patients show that the national 1-year relative survival rates for all cancer sites vary from 58.2% to 81.1% [17], with large variation even within EUROCARE five main European regions.

The World Health Organisation has recommended that, to increase cancer survival, reduce mortality and improve quality of life, national health authorities should aim to reduce late presentation [18]. However, there is a substantial challenge in deciding where and how to achieve more timely cancer diagnosis [19]. In countries where poorer one-year cancer survival rates suggest that late diagnosis may be an important factor, it is unclear whether this is due to patients presenting later to health-care, whether they are not being referred quickly enough by those in primary care, or whether they are not being managed and investigated efficiently in secondary care [1].

Primary care plays a key role in the timely diagnosis of cancer [20–22] and primary care practitioners (PCPs) are perceived by patients as having a crucial place in cancer detection [23, 24]. However, general practitioners (GPs) and other PCPs have the challenge of identifying those patients that do have cancer among the many patients presenting with symptoms that can be similar for benign and for malignant diseases [20, 25]. When patients with cancer present without ‘red-flag’ symptoms, how the PCP acts is likely to depend on how their health system is organised [26].

It has been suggested that PCPs’ knowledge of their patients can be used to improve health service design [27], and that incorporating their knowledge of their patient populations could improve the development of better approaches to earlier cancer recognition and referral [28]. PCPs occupy a key role in detecting cancer and in the cancer diagnostic process [29], and there has been a call for research in this area [7]. While their knowledge of their own health systems could potentially help to improve the planning of more effective approaches to earlier cancer recognition and referral, their perceptions have not been previously studied, nor have the between-country differences in their recommendations been evaluated.

The Örenäs Research Group (ÖRG), a European group of primary care researchers that studies the primary care factors that relate to cancer survival, therefore decided to elicit the views of GPs and other PCPs from across Europe on how they thought the timeliness of cancer diagnosis could be improved, and how these varied across different European countries. The ÖRG’s previous research has identified PCPs’ views on the factors that could improve timeliness of cancer diagnosis [30], and the aim of this analysis was to identify and characterise clusters of countries whose PCPs perceive the same factors as being important in improving the timeliness of cancer diagnosis.

## Methods

### Design

This cluster analysis of qualitative data is part of a broader online survey conducted in 25 Örenäs group centres in 20 European countries between November 2015 and December 2016. The survey study protocol has been already published [31] as well as the results of a thematic analysis of the survey qualitative data [30].

There were participants in Bulgaria, Croatia, Denmark, England, Finland, France, Germany, Greece, Israel, Italy, Netherlands, Norway, Poland, Portugal, Romania, Scotland, Slovenia, Spain, Sweden and Switzerland. Local study leads were asked to either gain ethical approval or obtain a statement that formal ethical approval was not needed in their jurisdiction.

### Participants and recruitment

Participants were PCPs in the 25 centres of the Örenäs Research Group members. PCPs in training were excluded from the study.

Each ÖRG local lead was asked to email a survey invitation to the PCPs in their local health district, and to recruit at least 50 participants. With this large sample size, we expected to recruit a sufficiently varied sample with regards to gender, years since graduation, site of practice (urban, rural, remote), and size of practice. There was no maximum limit to the number of participants. If the minimum number of participants was not reached, local leads were asked to use a snowballing method to increase the required sample size [32]. In six countries (Denmark, Norway, Portugal, Romania, Slovenia, Sweden), the invitation was distributed to a national sample.

### Measurements

A questionnaire was designed after literature review in order to identify PCPs’ management decision-making when faced with a patient with symptoms that could be due to cancer. The questionnaire also contained several statements about how the organisation of their health system affected their decision to refer patients that could have cancer for further investigation or specialist consultation. Respondents were asked to give their level of agreement on each statement. Finally, an open-ended question was included: ‘How do you think the speed of diagnosis of cancer in primary care could be improved?’ The answers to this question are the qualitative data used in this study.

The questionnaire was piloted twice, first by 16 then by 49 PCPs in 16 Örenäs Research Group member countries. No changes in the questionnaire were needed after piloting. The questionnaires were translated into local languages when English was not the country's official language. Standardised translation, back-translation to assess semantic and conceptual equivalence, and cultural adaptation [33] were carried out [34]. This resulted in questionnaires that had been translated and adapted for the 20 countries in the study. These were put online using Survey Monkey; on-line surveys have been successfully used in research involving cancer care professionals [35].

### Identification of themes

Main themes were identified using thematic analysis [36]. The phases of analysis included coding, followed by the identification of themes. Two researchers independently coded the free text data from three countries and compared their coding for inconsistencies and agreement. MH then coded the data from the other countries, using computer assisted qualitative data analysis Software (MaxQDA 11) to assist this process. A third researcher (ME) independently evaluated the codes and themes, after which MH and ME compared their thematic analyses for inconsistencies and agreement. Team members from seven participating countries then independently considered the themes, discussed these and came to a consensus over the course of two meetings. Apart from SH, all of the team involved in the coding and thematic analysis were experienced general practitioners who were also active in primary care research. SH was a Masters Psychology student.

### Frequency of themes by country

The number of times per country that an item in a theme was mentioned by a PCP was entered into a spreadsheet. These numbers were converted into percentages per country, i.e. the percentage of responses for each individual country in which each of the themes were mentioned. We drew a heatmap to give a graphical comparison of the key theme frequencies by country.

### Cluster analysis

Cluster analysis was performed to identify clusters (groups) of countries that shared similar patterns (i.e. perceived the same themes as most important). *k*-means clustering is a method used to automatically partition a data set into *k* groups. It selects *k* initial cluster centres and then iteratively refines the process so that a) each instance, *d*_*i*_, is assigned to its closest cluster, and b) each cluster centre, *Cj*, is updated to be the mean of its constituent instances [37].

Finally, we performed comparisons between groups to characterise each of the clusters, using one-way ANOVA and Bonferroni post-hoc testing to explore differences between them. SPSS software was used and a significance level of 0.05 was adopted.

## Results

### Sample characterisation

A total of 1,830 PCPs completed the questionnaire. All participating centres received at least 50 responses, with a median of 72 respondents per country. In all, 1,351 PCPs gave an answer to the final, open-ended survey question on how the speed of diagnosis of cancer in primary care could be improved, with a median of 46 per country. The demographic distributions of the PCPs answering this question are shown in Table 1.

**Table 1:** Demographic distribution of PCPs who responded to the open-ended question

Characteristic	n (%)
***Total***	1,351 (100)
***Country***	
Bulgaria	45 (3.3)
Croatia	42 (3.1)
Denmark	71 (5.3)
England	25 (1.9)
Finland	39 (2.9)
France	35 (2.6)
Germany	31 (2.3)
Greece	50 (3.7)
Israel	42 (3.1)
Italy	52 (3.8)
Netherlands	84 (6.2)
Norway	46 (3.4)
Poland	103 (7.6)
Portugal	46 (3.4)
Romania	132 (9.7)
Scotland	55 (4.1)
Slovenia	52 (3.8)
Spain	332 (24.6)
Sweden	55 (4.1)
Switzerland	15 (1.1)
***Gender***	
Female	833 (61.6)
Male	513 (38.0)
Not stated	5 (0.4)
***Years since graduation***	
< 10	192 (14.2)
10–19	356 (26.4)
20–29	416 (30.8)
30–39	336 (24.9)
40 or over	47 (3.5)
Not stated	4 (0.3)
***Site of practice***	
Urban	816 (60.4)
Rural	314 (23.2)
Island	25 (1.9)
Mixed	194 (14.4)
Not stated	2 (0.1)
***Number of doctors in practice***	
1–2	337 (24.9)
3–5	344 (25.5)
6–9	290 (21.5)
10 or more	374 (27.7)
Not stated	6 (0.4)

### Distribution of main factors to improve timeliness of cancer diagnosis per country

We identified eighteen themes organising the content of the responses to this question. The list of themes, with the number of times that PCPs mentioned each theme, is shown in Table 2.

**Table 2:** List of themes and number of times they are mentioned by a PCP

	n (%)
Access to tests	357 (22.6)
Access to specialists	249 (15.8)
PCP knowledge skills & attitudes	168 (10.7)
Screening	139 (8.8)
Patient and Population issues	129 (8.7)
PCP workload	110 (7.0)
Finances, quotas & limits	78 (5.0)
Guidelines and protocols	59 (3.7)
PCP & practice issues	52 (3.3)
Not a problem/good enough/can't be made quicker	47 (3.0)
PCP/specialist relationship	44 (2.8)
Prevention	38 (2.4)
Miscellaneous	26 (1.7)
No opinion	11 (0.7)
PCP/national relationships	10 (0.6)
Specialist knowledge, skills & attitudes	10 (0.6)
Research/work needed	8 (0.5)
Unhappy with the survey	8 (0.5)
PCP responsibility	5 (0.7)
Miscellaneous	26 (1.7)
Meaning unclear	26 (1.7)
Total	1574

### Comparison of key themes by country

**Fig. 1:**
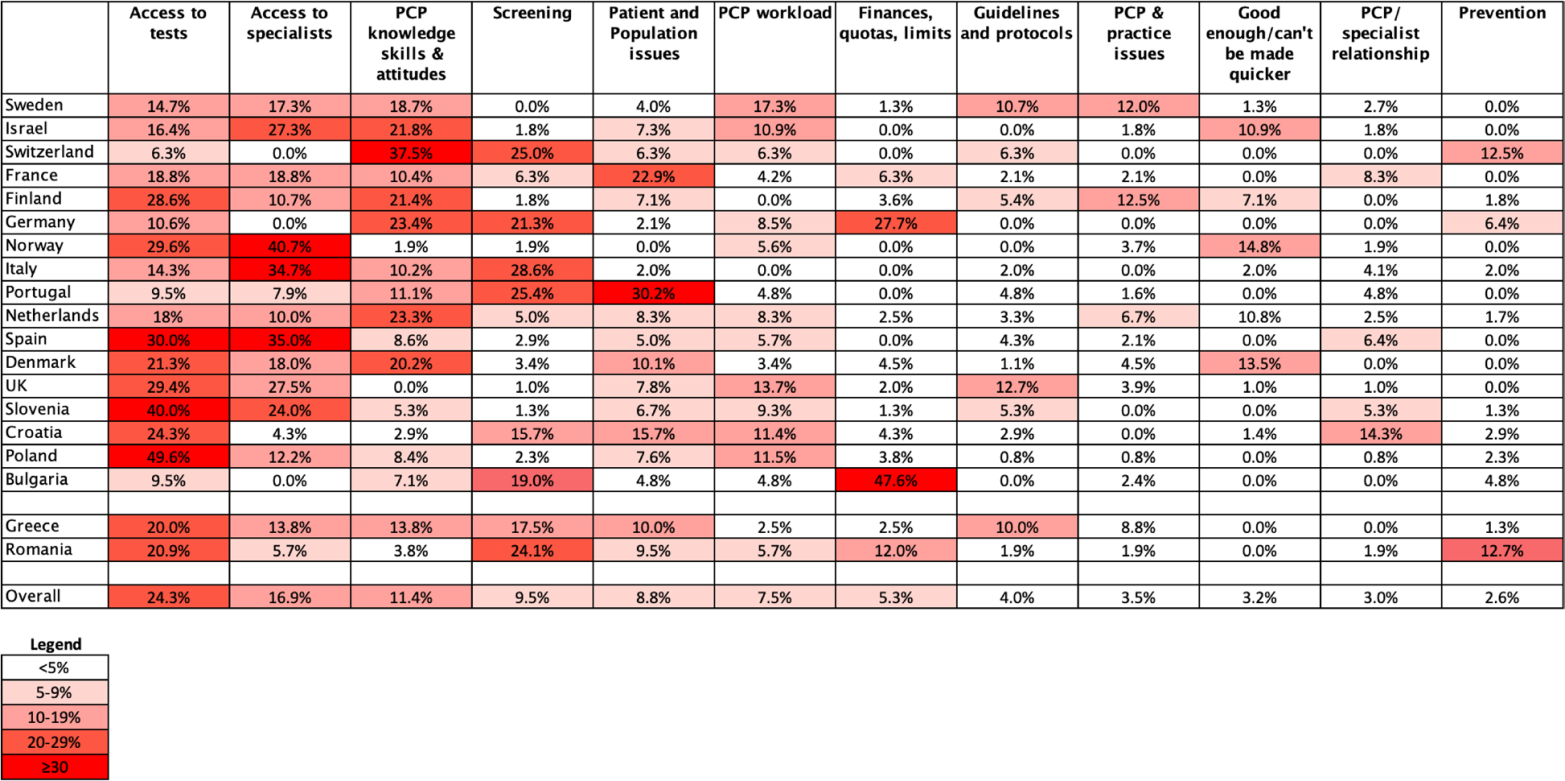
Heatmap comparing how frequently key themes are mentioned by PCPs in each country. Boxes show the percentage of times per country that an item in a theme was identified by a PCP. The columns are ordered by overall frequency; the rows are in descending order of 1-year cancer survival, with the exception of two countries that were unable to provide these data (Greece and Romania)

A heatmap comparing how frequently key themes are mentioned by PCPs in each country is shown in Fig. 1.

### Identification of clusters of countries that perceive the same factors as most important in improving the timeliness of cancer diagnosis

**Fig. 2:**
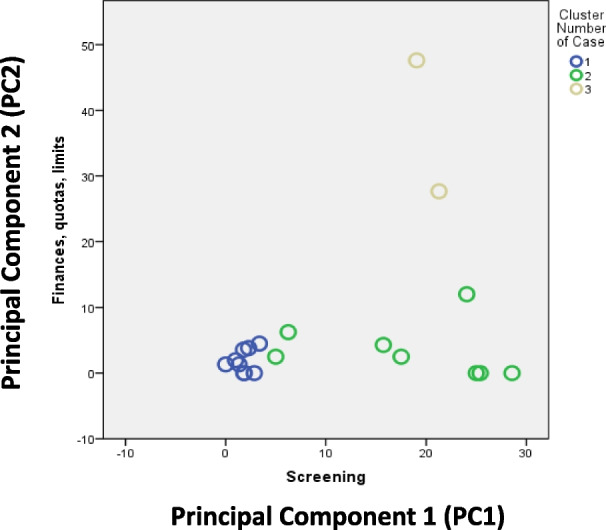
Cluster plot using *k*-means

*k*-means clustering identified three groups of countries that perceived the same factors as most important in improving the timeliness of cancer diagnosis. Cluster 1 included Sweden, Israel, Finland, Norway, Spain, Denmark, the United Kingdom (UK), Slovenia and Poland; Cluster 2 included Switzerland, France, Italy, Portugal, Netherlands, Croatia, Greece and Romania; and Cluster 3 included Germany and Bulgaria (Fig. 2).

### Characterisation of country clusters based on the most important factors to improve the timeliness of cancer diagnosis

There were significant differences between clusters regarding the importance of: access to tests (*p* = 0.010); access to specialists (*p* = 0.014), screening (*p* < 0.001) and finances, quotas & limits (*p* < 0.001) (Table 3).

**Table 3:** Characterisation of the country clusters

Dependent Variable	ANOVA	Bonferroni post-hoc test				
	*p*	Comparison between clusters		*p*	Lower Bound	Upper Bound
Access to tests	0.010*	1	2	0.030*	1.03	23.79
			3	0.044*	0.45	37.08
PCP knowledge skills and attitudes	0.855	1	2	1.000	-15.50	10.87
			3	1.000	-24.67	17.75
Patient and population issues	0.070	1	2	0.132	-15.39	1.54
			3	1.000	-10.87	16.36
Access to specialists	0.014*	1	2	0.090	-1.43	24.92
			3	0.026*	2.44	44.83
Screening	< 0.001*	1	2	0.000*	-24.40	-8.85
			3	0.004*	-30.86	-5.84
PCP workload	0.366	1	2	0.492	-2.67	9.09
			3	1.000	-7.49	11.42
Finances, quotas & limits	< 0.001*	1	2	1.000	-7.63	4.41
			3	0.000*	-45.49	-26.12
Guidelines and protocols	0.331	1	2	1.000	-4.59	5.23
			3	0.448	-3.42	12.37
PCP and practice issues	0.431	1	2	0.953	-3.14	7.08
			3	0.854	-4.81	11.62
Not a problem/good enough/can't be made quicker	0.239	1	2	0.480	-2.95	10.19
			3	0.570	-5.16	15.97
PCP/specialist relationship	0.229	1	2	0.621	-6.91	2.35
			3	1.000	-5.25	9.65
Prevention	0.090	1	2	0.181	-8.18	1.13
			3	0.285	-12.46	2.52

^*^p < 0.05

**Fig. 3:**
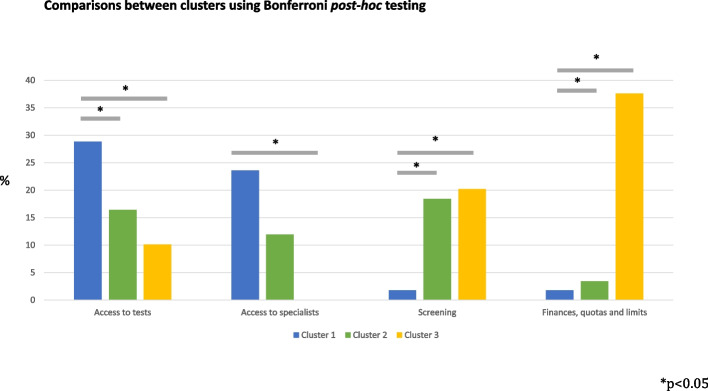
Comparisons between clusters using Bonferroni *post-hoc* testing. Bonferroni testing was used to explore significant differences between groups, with regards to the four variables that tested *p* < 0.05 in one-way ANOVA

While PCPs from countries in Cluster 1 valued more access to tests and specialists (and placed less value on screening and financial support), Cluster 3 shows exactly the opposite pattern. PCPs from countries in Cluster 2 highly valued access to tests and specialists, as well as screening programmes, but attributed a lower importance to financial aspects as strategies to improve timeliness of diagnosis. These comparisons are displayed in Fig. 3.

An overview on how these four themes are reported by country and cluster is provided in Table 4.

**Table 4:**
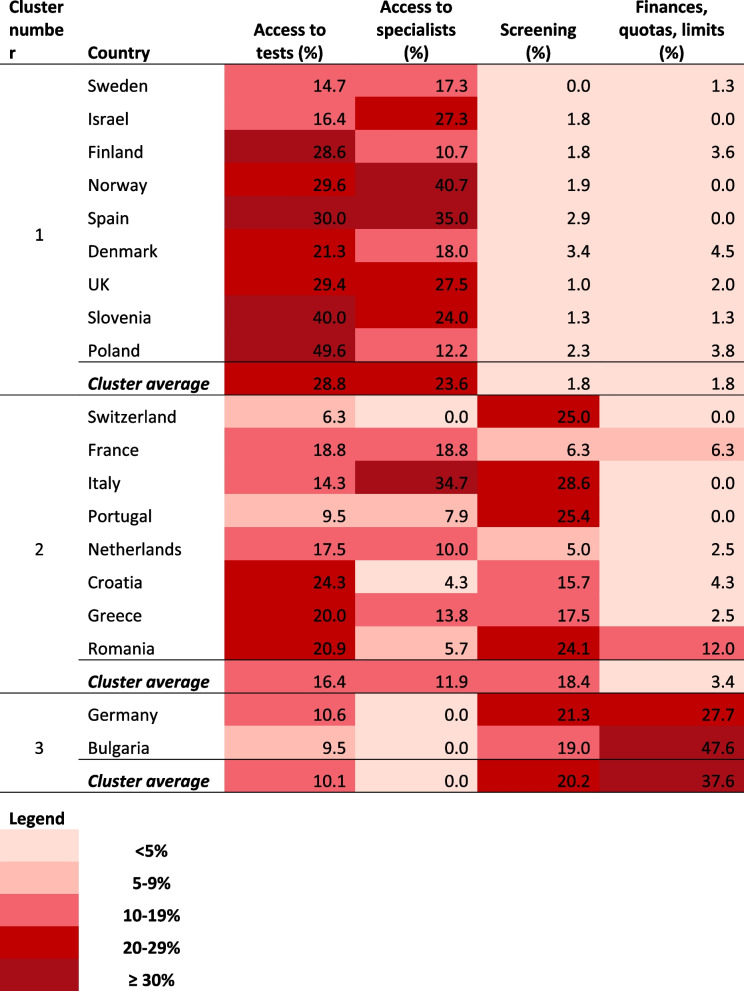
Distribution of significant themes by country and cluster

## Discussion

### Principal findings

Our analysis suggests that countries can be grouped into three clusters, based on PCPs’ perceptions about the strategies needed to improve the speed of cancer diagnosis. Significant differences were observed between these clusters regarding the relative importance of four themes: access to tests; access to specialists; screening and finances, quotas & limits. Countries in Cluster 1 (Sweden, Israel, Finland, Norway, Spain, Denmark, the UK, Slovenia and Poland) particularly value access to tests and specialists (and value screening and financial support less). Those in Cluster 3 (Germany and Bulgaria) show the opposite pattern. Countries in Cluster 2 (Switzerland, France, Italy, Portugal, Netherlands, Croatia, Greece and Romania) especially value screening programmes, and access to tests and specialists, while attributing a lower importance to financial aspects.

### Interpretation of the results

PCPs’ priorities for improving their management of patients that could have cancer are likely to reflect their views on the many non-clinical factors that affect their decision-making in these patients. Important explanatory factors may include levels of gate-keeping responsibility, funding systems, access to special investigations, fear of litigation and relationships with specialist colleagues [26]. Similarities across some countries may explain the clustering that we have identified. Differences between the clusters may be due to the varying impacts of these factors from country to country. Some factors, for example inequalities in implementation of cancer screening, could result from differing resources available for health care: there is a nine-fold variation in health care spending per capita across European countries [38], as well as differences in the quality of health care systems [39].

We can classify the factors that may explain the clustering by three different levels: national level factors, health system organisational issues, and factors affecting individual PCPs and their practices.

On the national level, variations in countries’ population sizes and densities, Gross National Products (GNP), proportions of GNP spent on health, and specifically on primary and preventative care, could be major factors. Differences in physical distances between outlying regions and first-tier secondary care facilities in a country may also be important. National differences in demographics, such as ethnicity and genetic predispositions, average age of the population, and percentage of the population over 65, may also be relevant. Varying health behaviour patterns across different countries (levels of obesity, differences in diet, alcohol use, smoking and exercise) could also be important, possibly modifiable, factors influencing the different clusters.

Differences in health system organisation have multiple possible levels of effect on the clustering that we identified. There may be variations in the extent to which electronic health records are used in primary and secondary care, and whether these systems are interlinked. Some countries have national cancer screening programmes, and these may vary in their coverage, and how they are financed and monitored. There may also be national differences in the percentage of healthcare expenditure that is ‘out of pocket’, waiting times to see specialists, and the extent to which patients can consult specialists or obtain advanced imaging if they pay more.

PCP- and practice-level factors that may explain the clustering include the status of primary care in each country, and the proportion of primary care is provided by doctors who have trained as GPs’. Other relevant issues include the extent to which the quality of PCPs is monitored, whether they are rewarded or punished for spending money on patient testing and specialist referrals, and whether they are permitted to request special tests without a specialist approval. There may also be variations in how much time they have in their consultations with patients, and whether they have a convenient and timely method to consult with their specialist colleagues.

### Comparison with other studies

The results of our study should be seen in the context of health care as a complex system whose elements interact in a non-linear way, often producing unexpected results [40].

Many of the countries in Clusters 1 and 2 have been identified as having a ‘GP as gatekeeper’ system [41], and it may be that their PCPs’ recognition that this creates a barrier to their patients explains why those PCPs particularly value, and therefore prioritise, access to specialists.

The PCPs in the Cluster 2 countries identified cancer screening as a priority, while access to tests, as well as access to specialists, were given less weight. This may be because, at the time of our survey, population-based cancer screening had not been fully implemented in most of those countries [42].

Bulgaria, in Cluster 3, has one of the lowest expenditures on primary care as a percentage of total healthcare expenditure [41], and this may explain its PCPs’ prioritisation of finances and quotas. Also, in previous published results of this study [43], PCPs in Germany and Bulgaria strongly agreed with the survey statement ‘We have a budget or quota for diagnostic tests’, and it may be that this results in financial constraints on investigation requests. However, unlike Bulgarian doctors, German doctors had high levels of agreement with the item ‘Referring or not referring does not affect me at all financially’. Bulgarian doctors had high levels of agreement for the statement that ‘Seeing a specialist can be a problem for some of my patients because of the financial costs to them’, but this was not the case for German doctors.

A narrative review investigated the extent to which health care systems influence the speed of cancer diagnosis in six well-developed countries. This was unable to establish a causal relationship between health care system characteristics and cancer outcomes. No differences were found in regulation, financing, patient list, gate-keeping role, direct access to secondary care, or the degree of comprehensiveness of primary care services [44].

The key themes identified in our study map across to those of a survey of GPs in Ireland, which identified that barriers to early cancer diagnosis included lack of direct GP access to diagnostic tests, difficulties with referral to secondary care, poor clarity relating to cancer screening, unequal patient access to secondary services, and a need for further training and guidelines [45]. GPs’ views on the importance of closer links between primary and secondary care were identified in that study, as well as in a UK study [24]. An Australian study of GPs’ perceptions of their role in cancer care found that all respondents valued better GP-specialist communication. They also identified a need to reduce system barriers and workforce pressures in general practice [29].

In a survey of GPs’ in England, fragmentation of the health service, continuity of care between primary and secondary health services, and staff working boundaries were found to be important [46]. In contrast to the findings of our study, GPs in England perceived there to be an excess of guidelines. However, in another UK study, guidelines seemed to have made little difference to the proportion of cases selected for referral on the fast-track pathway [47].

The need for healthcare systems to support PCPs’ quick and easy access to investigations has been proposed before [48]. While, in the UK, investigation in primary care has been linked with later referral for specialist assessment, reducing the waiting time for tests would be expected to shorten the primary-care intervals associated with investigation use [49]. Despite this, another study found that some patients received a delayed cancer diagnosis even when they had presented with typical cancer symptoms to a GP with access to relevant diagnostic tests [50].

### Strengths and limitations of this study

Our sample size was large and diverse, with 1,351 participants from 20 countries. Participants varied in terms of years of clinical practice, gender, and site and size of practice. As previously published, towards the end of the analysis, no new themes emerged and data saturation was achieved [30]. Variation in geography, health systems and levels of healthcare spending was ensured by including four participating countries from each of the Central, Eastern, Northern, Southern and Western European geographical areas.

Although this was a diverse sample, it was not a randomised sample, so there could have been selection bias. It is also important to note that there were differences in the number of responses in each country, with a potential impact on the representativeness of responses in countries with low response rates. The questionnaire only included a single, short question that related directly to our research question. However, it may be that this format prompted participants to focus on writing down what, for them, were the most important points.

Neither patients, secondary care nor other primary care health professionals were included in the survey, and these groups may have had other insights to offer.

### Implications for practice and policy

Understanding how countries’ characteristics impact their PCPs’ views on priority areas for improving timeliness of cancer diagnosis can lead to the development of more tailored guidelines at national and European level, with the potential to reduce inequalities in delivery of care. Those responsible for the organisation of healthcare in their countries will be able to identify which of this study findings are particularly relevant in their own jurisdictions. Some recommendations, for instance health education campaigns and development of relevant guidelines, may need central direction, though with the input of PCPs. Others, for example improving the way PCPs communicate with secondary care specialists, and PCPs’ ability to access cancer-specific tests, may need local agreement. Aspects such as PCPs’ own communication skills, their own accessibility to patients and their CME, are more likely to be under PCPs’ own control. However, consideration of how funding is best allocated is crucial if PCPs and their health systems are to make these changes.

Our results are purely descriptive, and the underlying reasons for the separation between clusters need to be explored in further work. In particular, future research should focus on whether there are within-country differences in the organisation of primary care that affect PCPs’ views. For example, there are within-country differences in access to investigations and fast-track referral initiatives [43], and the levels of population-based colorectal and cervical cancer screening [42].

Further work is needed to help identify which recommendations are most relevant to different existing models of healthcare, for example as to whether some are particularly relevant to systems in which the GP acts as a ‘gatekeeper’, or to those in which PCP practices are typically large, or more often small. A longitudinal study would give evidence on the trends in PCP opinions and how they are impacted by changes in health policies and public health initiatives.

Future studies should adopt inclusive approaches to ensure that a diverse range of views from different stakeholders is included.

## Conclusions

Our study identified the key factors that European PCPs believe would improve the timeliness of cancer diagnosis. Three distinct clusters of European countries are driven by four of these factors (‘access to tests’, ‘access to specialists’, ‘screening’, and ‘finances, quotas & limits’) in different ways. These insights pave the way to further analysis to understand what it is about these characteristics of the clusters that have produced these patterns, allowing healthcare systems to share best practice and to reduce disparities in timeliness of cancer diagnosis.

## Data Availability

The datasets used and/or analysed during the current study available from the corresponding author on reasonable request.

## Abbreviations

GP: General practitioner
ÖRG: Örenäs Research Group
PCP: Primary Care Practitioner
